# Fungal Endophthalmitis Associated with Compounded Products

**DOI:** 10.3201/eid2002.131257

**Published:** 2014-02

**Authors:** Christina A. Mikosz, Rachel M. Smith, Moon Kim, Clara Tyson, Ellen H. Lee, Eleanor Adams, Susanne Straif-Bourgeois, Rick Sowadsky, Shannon Arroyo, Yoran Grant-Greene, Julie Duran, Yvonne Vasquez, Byron F. Robinson, Julie R. Harris, Shawn R. Lockhart, Thomas J. Török, Laurene Mascola, Benjamin J. Park, for the Fungal Endophthalmitis Outbreak Response Team

**Affiliations:** Centers for Disease Control and Prevention, Atlanta, Georgia, USA (C.A. Mikosz, R.M. Smith, Y. Grant-Greene, B.F. Robinson, J.R. Harris, S.R. Lockhart, T.J. Török, and B.J. Park);; Los Angeles County Department of Public Health, Los Angeles, California, USA (C.A. Mikosz, M. Kim, C. Tyson, and L. Mascola);; New York City Department of Health and Mental Hygiene, New York City, New York, USA (E.H. Lee);; New York State Department of Health, Albany, New York, USA (E. Adams);; Louisiana Office of Public Health, New Orleans, Louisiana, USA (S. Straif-Bourgeois);; Nevada Division of Public and Behavioral Health, Carson City, Nevada, USA (R. Sowadsky);; Indiana State Department of Health, Indianapolis, Indiana, USA (S.Arroyo);; Illinois Department of Public Health, Springfield, Illinois, USA (Y. Grant-Greene);; Colorado Department of Public Health and Environment, Denver, Colorado, USA (J. Duran);; City of El Paso Department of Public Health, El Paso, Texas, USA (Y. Vasquez)

**Keywords:** eye infections, fungal, mold, endophthalmitis, disease outbreaks, Franck’s, *Fusarium incarnatum-equiseti*, *Bipolaris hawaiiensis*, fungi, compounding

## Abstract

Fungal endophthalmitis is a rare but serious infection. In March 2012, several cases of probable and laboratory-confirmed fungal endophthalmitis occurring after invasive ocular procedures were reported nationwide. We identified 47 cases in 9 states: 21 patients had been exposed to the intraocular dye Brilliant Blue G (BBG) during retinal surgery, and the other 26 had received an intravitreal injection containing triamcinolone acetonide. Both drugs were produced by Franck’s Compounding Lab (Ocala, FL, USA). *Fusarium incarnatum-equiseti *species complex mold was identified in specimens from BBG-exposed case-patients and an unopened BBG vial. *Bipolaris hawaiiensis* mold was identified in specimens from triamcinolone-exposed case-patients. Exposure to either product was the only factor associated with case status. Of 40 case-patients for whom data were available, 39 (98%) lost vision. These concurrent outbreaks, associated with 1 compounding pharmacy, resulted in a product recall. Ensuring safety and integrity of compounded medications is critical for preventing further outbreaks associated with compounded products.

Endophthalmitis is inflammation of the intraocular cavities and is often caused by infection (*1*). Exogenous endophthalmitis is a complication for ≈2–4 per 10,000 intravitreal injections (*2*,*3*) or pars plana vitrectomies (*4*). Most infections are bacterial; fungal infections are rare (*5*–*7*). The clinical course of fungal endophthalmitis is frequently prolonged and is associated with poor outcomes; vision loss is not uncommon (*5*,*8*–*10*). We describe 2 concurrent multistate outbreaks of fungal endophthalmitis associated with intraocular use of contaminated products labeled as sterile from a single compounding pharmacy.

## Methods

### Initial Epidemiologic Investigation

On March 5, 2012, the Healthcare Associated Infections Program of the California Department of Public Health was alerted to a cluster of 9 cases of fungal endophthalmitis, all among patients at the same Los Angeles County ambulatory surgical center who had undergone pars plana vitrectomies during October–December 2011. Two patients had histopathologic evidence of fungal hyphae in intraocular eye specimens; the others had a clinical diagnosis of fungal endophthalmitis. The initial investigation, led by the Los Angeles County Department of Public Health, demonstrated that all 9 patients had been exposed to a single lot of Brilliant Blue G dye (BBG), used to stain epiretinal membranes during vitrectomies. Although BBG is not approved by the Food and Drug Administration (FDA) for human use, it is increasingly being used in certain ocular procedures (P. Dugel, pers. comm.). The BBG implicated in the cases reported here was produced on August 23, 2011, at Franck’s Compounding Lab (Franck’s), a compounding pharmacy in Ocala, Florida, USA, which distributes products throughout the United States.

During the investigation, cases were defined as laboratory-confirmed or suspected fungal endophthalmitis among patients who had undergone vitrectomy at the Los Angeles County ambulatory surgical center during October 2011–January 2012, when the implicated BBG lot was in use. No additional cases were identified. A case–control study was conducted among 42 vitrectomy patients (the 9 case-patients and 33 control participants) at the same ambulatory surgical center (*11*). Information was obtained with regard to the operating surgeon, patient sex, and preoperative and intraoperative medications commonly used for vitrectomies at the Los Angeles County ambulatory surgical center. The only factor significantly associated with case status was Franck’s BBG (odds ratio ∞, p<0.001). Microbiological testing, conducted at a commercial laboratory, of an unopened BBG vial from the same lot yielded the environmental mold *Fusarium.* These data collectively turned the investigation focus on Franck’s products.

### Multistate Investigation

Franck’s records indicated that BBG from the contaminated lot was shipped to 22 facilities in 15 states. On March 9, 2012, Franck’s recalled all lots of BBG. That same day, the Centers for Disease Control and Prevention (CDC) and state and local health departments initiated a multistate investigation to identify additional cases of fungal endophthalmitis associated with invasive ocular procedures, identify the outbreak source, and prevent further exposures and illness.

The multistate investigation defined a probable case as ophthalmologist-diagnosed fungal endophthalmitis occurring after an invasive ophthalmic procedure performed on or after August 23, 2011; each affected eye was counted separately as a case. A case was considered confirmed after laboratory identification of fungi from eye specimens by culture, genetic sequencing, or histopathologic examination, at local hospitals, public health laboratories, or CDC.

On March 26, 2012, CDC was notified of a patient in whom fungal endophthalmitis had developed during February 2012 after an intravitreal injection of triamcinolone acetonide (triamcinolone) at a New York ophthalmology practice. Triamcinolone is a corticosteroid used to treat a variety of ophthalmic conditions. Three additional patients with suspected fungal endophthalmitis were identified at the practice; all 4 had received intravitreal injections of triamcinolone from a single lot manufactured at Franck’s on November 4, 2011. Preliminary CDC laboratory testing identified *Bipolaris hawaiiensis*, a rare environmental mold infrequently described as a human pathogen, in ocular specimens from these case-patients. Because Franck’s invoices indicated that triamcinolone from the same lot had been shipped to 5 ophthalmology practices in 4 states, the investigation was expanded. On March 31, 2012, Franck’s recalled this lot of triamcinolone.

### Case Finding

Case finding was conducted through postings to Epi-X (www.cdc.gov/epix/), a secure notification network for public health professionals. Email messages were sent to ClinMicroNet (www.asm.org/index.php/online-community-groups/listservs), a network of clinical microbiology laboratories; to academic microbiology laboratories known from previous ophthalmic disease outbreaks (*12*–*14*); and to members of 2 major ophthalmology professional societies. CDC also reviewed available Franck’s internal adverse event logs and sales records and contacted physicians listed in these reports.

### Laboratory Testing

CDC performed fungal cultures on case-patient specimens, including vitreous fluid, intraocular lenses, and intraocular swabs. CDC also performed confirmatory testing on fungal specimens from other laboratories, including a fungal isolate recovered by FDA from a BBG vial and fungal DNA isolated by an outside laboratory from a vitreous cassette specimen. Fungal isolates were identified by morphologic and DNA sequence analysis (*15*). *Fusarium* isolates were compared by multilocus sequence analysis (*16*); species of *Bipolaris* isolates were identified by examination of the ribosomal internal transcribed spacer region (*15*).

### Case–Control Analyses

To confirm that Franck’s products were associated with cases identified outside California and to evaluate the hypothesis that there were 2 separate outbreaks, both associated with Franck’s, we conducted 2 case–control studies. One case–control study was conducted for each fungal species outbreak (*Fusarium* infections associated with Franck’s BBG administered during vitrectomies and *Bipolaris* infections associated with Franck’s triamcinolone injected intravitreally); only species-confirmed cases (i.e., cases confirmed by culture) were included. A third case–control analysis separately examined probable cases. Control participants were well patients from the same clinical practice, matched 3:1 on case-patient procedure type and week of the procedure during which case-patient exposure to recalled Franck’s BBG or triamcinolone first occurred. Medical charts were abstracted by using a standardized form. Case-patients were followed up after exposure to either of the 2 Franck’s products for either 6 months or until documented resolution of infection, whichever occurred first; vision loss in this analysis reflects documentation of reduced visual acuity by the treating ophthalmologist at any point during this 6-month period and not necessarily permanent vision loss. Statistical analysis was conducted by using SAS version 9.2 (SAS Institute Inc., Cary, NC, USA); categorical variables were assessed by the Cochran-Mantel-Haenszel test, by using the matched quartets as a stratification factor; and 95% CIs and p values were calculated by using exact methods.

This investigation was considered to be an urgent public health response and thus was not considered to be research that required approval by an institutional review board or informed consent from involved patients. All patient names and protected health information were kept confidential.

## Results

As of March 22, 2013, a total of 47 cases among 45 patients had been identified in 9 states. Twenty-three cases were confirmed through histopathologic examination or microbiological testing; of these, infection with *F. incarnatum-equiseti* species complex mold was confirmed by culture for 7, and a different mold, *B. hawaiiensis*, was confirmed by culture for 9. The rest of the cases were confirmed only by histopathology. The definition of a probable case was met by 24 cases. Two case-patients were hospitalized; none died.

CDC confirmed *F. incarnatum-equiseti* species complex in an isolate derived from an unopened BBG vial from the implicated lot. *Fusarium* isolates from case-patients and the BBG vial were indistinguishable by multilocus DNA sequencing at 4 loci. *B. hawaiiensis* was confirmed in 10 isolates from 9 case-patients in 3 states.

### Case–Control Studies

Preliminary evidence indicated that there were 2 separate, concurrent outbreaks associated with 2 Franck’s products: *Fusarium* infections associated with BBG and *Bipolaris* infections associated with triamcinolone. Results of the several case–control analyses are as follows.

#### *Fusarium* Case Cluster

 This analysis included 6 confirmed case-patients and 18 control participants (Table 1). Only Franck’s BBG was significantly associated with *Fusarium*-confirmed fungal endophthalmitis (p = 0.002). All *Fusarium*-confirmed case-patients had been exposed to BBG. Female sex was also associated with case status (p = 0.05).

**Table 1:** Case–control results for species-confirmed fungal endophthalmitis cases, United States, 2012*

Variable	*Fusarium* spp. cluster				*Bipolaris* spp. cluster		
	Case-patients, no. (%), n = 6	Controls, no. (%), n = 18	mOR (95% CI)		Case-patients, no. (%), n = 7	Controls, no. (%), n = 18	mOR (95% CI)
Patient characteristic							
Female sex	5 (83)	4 (22)	12.0 (0.99–472.73), p = 0.05		3 (43)	11 (61)	0.61 (0.05–5.71), p = 0.94
Diabetes	3 (50)	7 (39)	1.50 (0.18–11.43), p = 0.98		6 (86)	4 (22)	13.33 (1.12–492.51), p = 0.04
Hypertension	3 (50)	7 (39)	1.40 (0.15–24.76), p>0.99		7 (100)	15 (83)	Undef (0.33–∞), p = 0.50
History of eye surgery or procedures	4 (67)	12 (67)	1.00 (0.11–12.77), p>0.99		4 (57)	16 (89)	0.25 (0.003–2.440), p = 0.28
Medications received							
Cyclopentolate	4 (67)	15 (83)	0 (0–6.33), p = 0.50		0	0	NC
Phenylephrine	3 (50)	15 (83)	0 (0–1.16), p = 0.13		0	0	NC
Tropicamide	0	0	NC		0	0	NC
Bupivacaine	0	4 (22)	0 (0–3.47), p = 0.50		0	0	NC
Atropine	0	4 (22)	0 (0–3.00), p = 0.38		0	0	NC
Lidocaine	1 (17)	7 (39)	0.20 (0.005–4.32), p = 0.59		2 (29)	5 (28)	1.00 (0.008–130.300), p>0.99
Tetracaine	2 (33)	10 (56)	0 (0–3.40), p = 0.56		3 (43)	3 (17)	Undef (0.16–∞), p = 0.50
Brilliant blue G dye†	6 (100)	2 (11)	Undef (3.47–∞), p = 0.002		0	0	NC
Cefazolin	0	1 (6)	0 (0–57.00), p>0.99		0	0	NC
Antimicrobial ophthalmic ointment	0	3 (17)	0 (0–5.81), p = 0.75		1 (14)	0	Undef (0.16–∞), p = 0.50
Vancomycin	2 (33)	6 (33)	1.00 (0.06–16.26), p>0.99		0	0	NC
Moxifloxacin	4 (67)	11 (61)	1.50 (0.07–91.71), p>0.99		0	3 (17)	0 (0–3.87), p = 0.50
Triamcinolone†	0	0	NC		7 (100)	2 (11)	Undef (3.68–∞), p = 0.001
Bevacizumab	0	0	NC		5 (71)	14 (78)	1.00 (0.008–130.30), p>0.99
Dexamethasone	0	3 (17)	0 (0–5.14), p = 0.84		0	10 (56)	0 (0–0.52), p = 0.02

*mOR, median odds ratio; Undef, undefined; NC, not calculated. †Manufactured by Franck’s Compounding Lab, Ocala, Florida, USA.

#### *Bipolaris* Case Cluster

Among 7 confirmed case-patients and 18 control participants in the study, Franck’s triamcinolone was significantly associated with *Bipolaris*-confirmed infection (p = 0.001); all *Bipolaris*-confirmed case-patients had received this product (Table 1). Diabetes mellitus was also statistically associated with case status (p = 0.04).

#### Probable Case Cluster

All probable case-patients had been exposed to either Franck’s BBG or triamcinolone; among 17 enrolled probable case-patients and 51 control participants, exposure to either product was the only factor significantly associated with case status (p<0.001; Table 2). No case-patients were exposed to both products.

**Table 2:** Case–control study results for probable fungal endophthalmitis cases, United States, 2012*

Variable	Case-patients, no. (%), n = 17	Controls, no. (%), n = 51	mOR (95% CI)	p value
Patient characteristic				
Female	10 (59)	25 (49)	1.45 (0.43–5.12)	0.68
Diabetes	10 (59)	20 (39)	1.77 (0.63–6.82)	0.28
Hypertension	10 (59)	30 (59)	1.00 (0.30–3.45)	>0.99
History of eye surgery or procedures	10 (59)	39 (76)	0.47 (0.14–1.71)	0.30
Medication received				
Cyclopentolate	0	8 (16)	0 (0–0.83)	0.07
Phenylephrine	8 (47)	28 (55)	0.33 (0.02–4.60)	0.52
Tropicamide	8 (47)	22 (43)	Undef (0.10–∞)	>0.99
Bupivacaine	8 (47)	25 (49)	0.83 (0.08–10.28)	>0.99
Atropine	5 (29)	7 (14)	3.67 (0.56–44.41)	0.22
Lidocaine	11 (65)	33 (65)	1.00 (0.21–5.61)	>0.99
Tetracaine	8 (47)	24 (47)	1.00 (0.17–5.33)	>0.99
Brilliant blue G dye or triamcinolone†	17 (100)	7 (14)	Undef (11.90–∞)	< 0.001
Cefazolin	5 (29)	19 (37)	0.33 (0.02–4.60)	0.52
Antimicrobial ointment	8 (47)	17 (33)	3.33 (0.48–262.41)	0.27
Vancomycin	2 (12)	3 (6)	Undef (0.16–∞)	0.50
Moxifloxacin	3 (18)	6 (12)	4.00 (0.14–196.39)	0.75
Bevacizumab	5 (29)	20 (39)	0.17 (0.003–3.20)	0.31
Dexamethasone	5 (29)	24 (47)	0.25 (0.04–1.48)	0.14

*mOR, median odds ratio; Undef, undefined. †Manufactured by Franck’s Compounding Lab, Ocala, Florida, USA.

### Case Summaries

**Figure 1:**
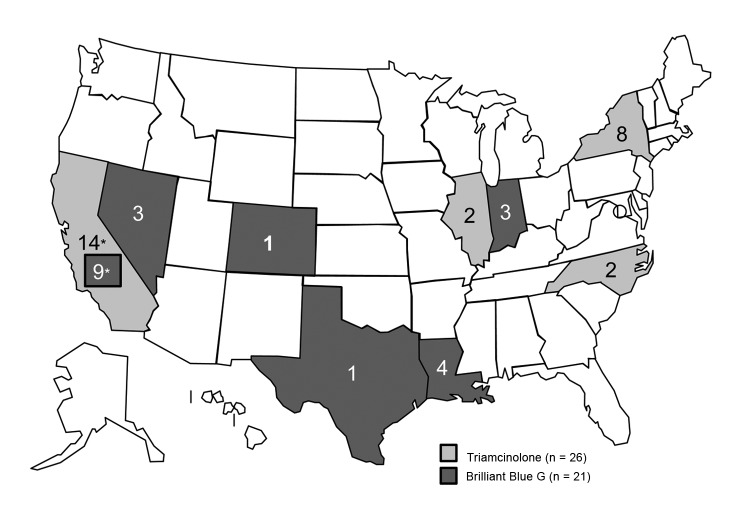
Confirmed and probable cases of postprocedural fungal endophthalmitis, by state, United States, 2011–2012. Infections occurred after exposure to a product from Franck’s Compounding Lab (Ocala, FL, USA), though March 2012, when the implicated product was recalled. *In California, cases were associated with exposure to each product.

With epidemiologic and laboratory evidence demonstrating 2 distinct outbreaks associated with 2 Franck’s products, all 47 cases could be sorted into 1 of 2 outbreak cohorts. Overall, 21 cases of fungal endophthalmitis were associated with BBG exposure and *Fusarium* mold and 26 cases were associated with Franck’s triamcinolone exposure and *Bipolaris* mold (Figure 1).

**Figure 2:**
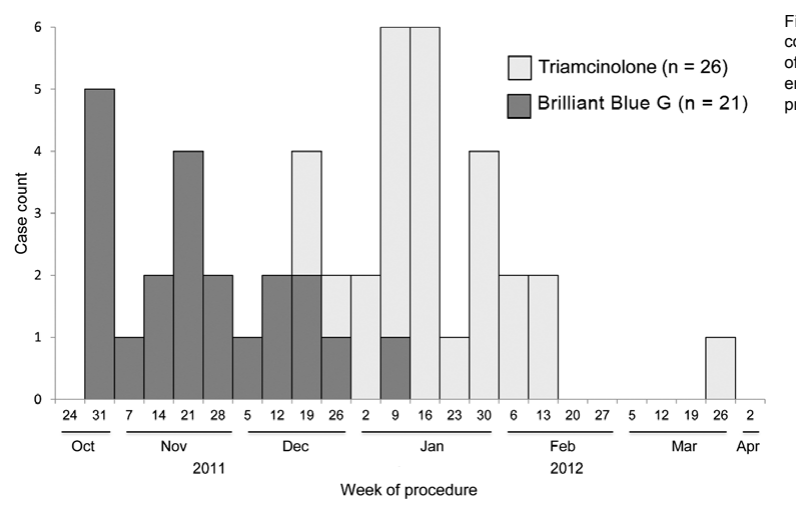
Epidemic curve of confirmed and probable cases of postprocedural fungal endophthalmitis, by week of procedure, United States.

Dates of symptom onset were difficult to ascertain because of the subacute onset of disease; Figure 2 reports case counts by date of procedure during which exposure to BBG or triamcinolone first occurred. In this outbreak, exposure to Franck’s BBG occurred primarily during late October–late December 2011, whereas exposure to Franck’s triamcinolone occurred during December 2011–March 2012.

#### BBG-associated Outbreak

Data were available for all 21 BBG-exposed case-patients (Table 3). Among these case-patients, 16 (76%) were women, median age was 69 years (range 58–86), 11 (52%) had a history of diabetes mellitus 11 (52%) had a history of hypertension, and 14 (67%) had undergone previous eye surgeries or procedures. All patients in this cluster reported unusual vision loss. Vitreous debris was noted during examination of 12 (57%) case-patients; hypopyon, eye inflammation, and fibrin were observed in 9 (43%), 10 (48%), and 12 (57%) case-patients, respectively. Eye pain was reported by 16 (76%) case-patients and floaters by 4 (19%).

**Table 3:** Clinical characteristics of fungal endophthalmitis case-patients, United States, 2012*

Characteristic	Exposure cluster†	
	Brilliant Blue G dye, n = 21	Triamcinolone, n = 19
Demographics		
Median age, y (range)	69 (58–86)	67 (53–77)
Female sex	16 (76)	8 (42)
Concurrent medical conditions		
Diabetes mellitus	11 (52)	15 (79)
Hypertension	11 (52)	15 (79)
History of prior eye surgery or procedures	14 (67)	13 (68)
Signs and symptoms		
Vision loss	21 (100)	18 (95)
Vitreous debris	12 (57)	10 (53)
Floaters	4 (19)	6 (32)
Inflammation	10 (48)	6 (32)
Pain	16 (76)	4 (21)
Hypopyon	9 (43)	3 (16)
Fibrin	12 (57)	1 (5)
Treatment		
Any antifungal treatment	17 (81)	19 (100)
Intravitreal amphotericin B	2 (12)‡	8 (42)
Intravitreal voriconazole	17 (100)‡	11 (58)
Oral voriconazole	6 (35)‡	11 (58)
Intravenous voriconazole	1 (6)‡	0
Topical voriconazole	1 (6)‡	0
Oral fluconazole	1 (6)‡	0
Combination of antifungal therapies	10 (59)‡	9 (47)
Outcome		
Median no. days from exposure to diagnosis (range)	78 (60–125)§	80 (35–185)
Additional surgeries required (range)	20 (95) (1–5)	16 (84) (1–6)
No documentation of resolved infection within follow-up period	3 (14)	8 (42)
Enucleations	0	2 (11)

*Data available for 40 case-patients and are presented as no. (%) unless otherwise indicated. †Manufactured by Franck’s Compounding Lab, Ocala, Florida, USA. ‡Calculated percentage out of 17 treated case-patients. §n = 18.

Antifungal therapy was received by 17 (81%) case-patients. Among all treated case-patients, 10 (59%) received combination antifungal therapy. The most common treatment was intravitreal voriconazole, received by all 17 (100%) treated case-patients (Table 3). Oral voriconazole was the next most common treatment, received by 6 (35%) treated case-patients, followed by intravitreal amphotericin B, received by 2 (12%) treated case-patients. The median time from exposure to diagnosis in this cluster was 78 days (range 60–125); 95% of patients required additional surgeries (e.g., vitrectomies) to treat their infections (Table 3). After 6 months, 3 (14%) case-patients did not have documentation of resolved infection.

#### Triamcinolone-associated Outbreak

Detailed data were available for 19 (73%) of 26 triamcinolone-exposed case-patients (Table 3). Among these case-patients, 8 (42%) were women, median age was 67 years (range 53–77), 15 (79%) had a history of diabetes mellitus, and 15 (79%) had a history of hypertension. Most case-patients had a history of previous eye surgery or procedures. Virtually all (18 [95%]) case-patients experienced vision loss. Vitreous debris was commonly noted on examination. Hypopyon, inflammation, and fibrin were also observed during examination of the affected eye, although with lower frequency than for patients in the BBG cluster (Table 3). Pain was reported by 4 (21%) case-patients and floaters by 6 (32%). Two patients had received bilateral injections with triamcinolone, and for each of these patients, infection subsequently developed in both eyes.

All case-patients in this cluster received antifungal therapy, most commonly with intravitreal (11 [58%]) or oral (11 [58%]) voriconazole (Table 3). Most case-patients received a combination of antifungal therapies. Intravitreal amphotericin B was received by 8 (42%) case-patients. Median time from exposure to diagnosis in this cluster was 80 days (range 35–185); 84% of case-patients required additional surgeries (Table 3). After 6 months, documentation of resolved infection was unavailable for approximately half (42%) of these case-patients; 2 required enucleation because of severe infection. Both enucleations were performed after the 6-month follow-up period and were not systematically reported. Additional data for these 2 case-patients are not available, and more enucleations or other unreported complications among case-patients are possible.

### Public Health and Regulatory Action

On May 3, 2012, CDC and state and local public health officials reported the outbreaks (*17*), publishing the name of Franck’s and advising against use of any of Franck’s sterile compounded products while the investigation was ongoing. On May 25, Franck’s suspended all sterile compounding services and announced a recall of all sterile compounded products distributed during November 21, 2011–May 21, 2012, in response to an FDA investigation that revealed fungal growth in Franck’s clean room, where sterile compounds were produced (*18*). Further details of the FDA findings are not publicly available.

## Discussion

Two concurrent outbreaks of fungal endophthalmitis were associated with 2 environmental molds contaminating 2 compounded medications labeled as sterile from the same compounding pharmacy. Together, these 2 outbreaks represent the largest reported outbreak of infectious endophthalmitis and one of the largest outbreaks in the United States attributed to contamination of a compounded medication.

Infections among case-patients were characterized by poor outcomes, including vision loss, as has been described for fungal endophthalmitis (*5*,*9*,*10*). Prolonged time to diagnosis probably contributed to these poor outcomes; diagnostic delay might be explained by the rarity of fungal endophthalmitis and the resulting unfamiliarity of some physicians with this diagnosis, the subacute nature of early infection, or the difficulty of distinguishing true infection from the occasionally observed postprocedural sterile inflammation (*19*). Female sex was associated with case status among patients in the BBG cluster; however, sex differences have not been reported for prior clusters of *Fusarium* endophthalmitis patients (*20*,*21*). The association of diabetes mellitus with case status among patients in the triamcinolone cluster probably reflects the fact that multiple patients received these injections for complications associated with diabetes or that diabetes might have predisposed the patients to infection after exposure.

Compounded medications are a vital part of the health care delivery system, but the role of BBG in this outbreak merits focused discussion. BBG is a triphenylmethane dye in the Coomassie dye family, commonly used for protein staining in biochemical analysis (*22*–*24*). BBG became popular among ophthalmologists for ocular surgery requiring dyes (P. Dugel, pers. comm.) because of its superior safety profile (*25*,*26*) and staining capabilities, compared with FDA-approved products (*27*,*28*). Although BBG is licensed and manufactured in several European countries, it is not FDA approved for any human use in the United States and cannot be manufactured by US pharmaceutical companies for clinical indications. Instead, BBG for clinical use in the United States is provided by compounding pharmacies. Whether providers who administered BBG or the patients who received it were aware that it was not FDA approved for human use is unknown. Regardless, greater transparency and improved education among involved parties as to BBG’s regulatory status (as a medication not approved by FDA for human use), particularly at the time of purchase, might have affected the extent of its use and, subsequently, the number of infections in these outbreaks.

This investigation had certain limitations. First, although the epidemiologic association between case status and triamcinolone exposure was strong, triamcinolone from the implicated Franck’s lot was unavailable for testing (all vials had been either opened or used in their entirety when the investigation began). Second, follow-up data through 6 months after the procedure or resolution of infection, whichever occurred first, was not available for all case-patients. Third, whether a lack of documented resolution of infection at the end of the 6-month follow-up period truly represented ongoing infection for all case-patients is unclear. Detailed treatment data, including dosages, were not systematically available, and some case-patients were still receiving treatment at the end of the 6-month follow-up period, which limited our ability to correlate treatment strategy with patient outcomes. Last, objective data on visual acuity (e.g., 20/20 scale measurements) were not available for all case-patients; because some degree of temporary, minor vision loss is not unusual after any invasive ocular procedure (P. Dugel, pers. comm.), we relied on the clinical judgment of treating ophthalmologists to define atypical postprocedural vision loss.

These outbreaks of fungal infections are not the first associated with contaminated compounded products; they preceded (by only a few months) a large multistate outbreak of *Exserohilum rostratum* infections associated with compounded methylprednisolone acetate (*29*). A 2002 outbreak of meningitis and sacroiliitis attributed to compounded corticosteroid injections contaminated with *Exophiala dermatitidis* was also reported (*30*). Unlike outbreaks associated with bacteria in compounded medications, those associated with fungi present unique challenges, because the insidious, prolonged nature of certain fungal infections might obscure an association with an earlier medication exposure. In addition, unlike bacteria, fungi are often difficult to isolate by culture; molecular diagnostic techniques, such as PCR, might be more useful, but their availability to the average clinician is limited. Furthermore, when directly inoculated into sterile spaces, these fungi can cause infections that are challenging to recognize, as we observed in these outbreaks, not only because they often involve environmental agents that are exceedingly rare causes of human illness but also because once instilled into a sterile space, they can cause disease in an uncommon anatomic area or in an unusual way. 

*Bipolaris* spp. are a common cause of chronic sinusitis among immunocompetent persons (*31*); however, before this outbreak, endophthalmitis with *Bipolaris* spp. had been described in limited case reports only (*32*,*33*). Similarly, fusariosis usually occurs in immunocompromised hosts; endophthalmitis has been reported but usually as a sequela of *Fusarium* keratitis (*10*,*34*) and not de novo, as in this outbreak. The rarity of the infections in these outbreaks provided an additional clue to clinicians that an atypical event had occurred, underscoring the crucial role of clinicians in recognizing unusual disease clusters and promptly reporting them to public health authorities for rapid epidemiologic investigation necessary to rule out widespread exposure.

Oversight of compounding pharmacies lies primarily with state pharmacy boards because compounding pharmacies are regulated as pharmacies despite the fact that some (e.g., Franck’s) produce large quantities of medications and distribute them across state lines. Federal oversight of compounding pharmacies has been limited and often contested. FDA had investigated Franck’s twice before these outbreaks. During 2004–2005, FDA inspected Franck’s for improper compounding practices, including compounding medications without a valid patient prescription (*35*), and during 2009, Franck’s veterinary compounding unit incorrectly compounded a nutritional supplement that resulted in the deaths of 21 polo horses (*36*). These investigations did not preclude Franck’s from continuing its operations. During 2010, FDA sought a federal injunction to compel Franck’s to cease veterinary compounding from bulk ingredients (*37*) but ultimately lost the court case (*38*). Enhanced regulatory authority toward compounding pharmacies with multiple infractions should be considered a part of efforts to improve compounded medication safety. Furthermore, greater transparency surrounding ongoing disciplinary action against a compounding pharmacy might empower clinicians to make more informed purchasing decisions. Enhanced oversight of compounding pharmacies that produce large quantities of medications, especially sterile products that are distributed nationwide, should be considered to ensure that mass-produced sterile compounded products are safe.

Clinicians should be aware that the availability of a compounded medication in the United States is not a guarantee of its quality or of FDA approval. Disclosure of a medication’s FDA approval status should be encouraged at all stages of purchase and use. This information might enable clinicians to make informed decisions about the medications they purchase for patient use and to educate patients about the status of medications to which they are exposed. Maintenance of the safety and integrity of sterile compounded drugs in the United States demands a thorough review and improvement of compounding pharmacy regulatory practices.
